# Assessing Benzene and TVOC Pollution and the Carcinogenic and Noncarcinogenic Risks to Workers in an Industrial Plant in Southeastern Romania

**DOI:** 10.3390/toxics12030187

**Published:** 2024-02-28

**Authors:** Sebastian-Barbu Barbeş, Alina Bărbulescu, Lucica Barbeș

**Affiliations:** 1 Doctoral School of Civil Engineering, Technical University of Civil Engineering Bucharest, 122-124 Lacul Tei Bvd., 020396 Bucharest, Romania; sebastian-barbu.barbes@phd.utcb.ro; 2 Department of Civil Engineering, Transilvania University of Brașov, 5 Turnului Str., 500152 Braşov, Romania; 3 Department of Chemistry and Chemical Engineering, Ovidius University of Constanța, 124 Mamaia Bd., 900112 Constanta, Romania; 4 Doctoral School of Biotechnical Systems Engineering, Politehnica University of Bucharest, 313, Splaiul Independenţei, 060042 Bucharest, Romania

**Keywords:** benzene, TVOC, statistical analysis, POT, HI, LRC

## Abstract

The article aims to analyze the pollution with Volatile Organic Compounds (VOC) emitted from the biggest refinery in Romania, using the daily and monthly series registered for two years in two sites on the industrial platform, and the carcinogenic and noncarcinogenic risks for workers at the industrial plant. Since the values of the basic statistics (minimum, maximum, and average) and outliers indicate that most recorded values exceed the maximum admissible limits established by law, the Peaks Over Threshold (POT) method was utilized to model the maximum values of the series and determine the return levels for benzene and total VOC (TVOC). Given the high values obtained for relatively short return periods, indicating potential danger for the workers, we assessed the noncarcinogenic and carcinogenic risks to benzene and TVOC exposure by computing the hazard index (HI) and lifetime cancer risk (LCR). The results indicate that 43.75% of the HI values are above 1, indicating a relatively high noncarcinogenic risk for different categories of workers. LRC indicates a high LRC for 93.75% of the workers in all considered categories exposed to TVOC.

## 1. Introduction

VOCs are substances with the property that at least a fifth of their weight is formed by vapor whose pressure exceeds 300 Pa at 20 °C [1]. They are pollutants whose presence in the atmosphere may impact the population and environmental health [2,3]. About 150 compounds are classified as VOCs. They are predominantly hydrocarbons with 4–12 carbon atoms (paraffin, olefins, and aromatic hydrocarbons), benzene and its derivatives, etc., which have a high carcinogenic potential [2,4]. Total volatile organic compounds, expressed as non-methane volatile organic compounds (NMVOCs), including aromatic hydrocarbons (benzene, toluene, ethyl-benzene, and ortho-, meta-, and para-xylene, etc.) with moderate volatility, are found in different concentrations in urban and suburban regions [5,6,7,8,9,10], shoreline areas [11], and industrial zones [12,13,14,15].

Scientists have proved that environmental air pollution impacts the population dramatically, increasing morbidity and mortality, with over 4.2 million related deaths estimated to occur annually [16,17,18,19,20]. Cheng et al. [21] show that VOCs can provoke irritation in the throat, nose, and eyes, headaches, shortness of breath, skin problems, fatigue, dizziness, and nausea. Prolonged or elevated exposures may lead to lung irritation, kidney and liver impairment, cancer, and damage to the central nervous system. The health effects induced by VOCs depend on the concentration levels and the extent of exposure to the pollutants. Cao et al. [22] assessed the health risks for residents in the surrounding petrochemical and industrial parks located in the YRD region of China by computing the Health Index (HI) and the lifetime cancer risk (LCR) of TVOCs.

Various investigations show that long-term exposure to benzene can cause hematological diseases, such as acute and chronic lymphocytic leukemia, acute myeloid leukemia, multiple myeloma, non-Hodgkin’s lymphoma, and aplastic anemia [23,24,25]. Benzene has also been reported to induce mammary cancer [26,27,28,29]. Benzene has been classified as a group 1A carcinogen [30] as its toxic effects on the hematopoietic system are well known [31]. Other well-researched VOCs include polycyclic aromatic hydrocarbons, formaldehyde, and benzo[a]pyrene, with their cancer-causing potential confirmed by comprehensive data [32,33,34]. One study [35] emphasized that exposure to vinyl chloride can provoke a relatively rare cancer, liver angiosarcoma [35]. It was indicated that 70–75% of the estimated cancer risk was attributable to exposure to polycyclic organic matter, formaldehyde, 1,3-butadiene, and benzene [36]. An experimental study on mice pointed to the carcinogenicity of ethylbenzene [37]. Only one experimental study evaluated the effects of human exposure to ethylbenzene for 10 years. No tumor was reported, but the period is too short for cancer latency [38].

Studies have demonstrated that the oil refining and processing industry significantly contributes to VOC emissions [39,40]. There is an estimation that most refineries in Europe release a considerable amount of VOCs into the atmosphere (150–6500 tons/year, respectively, 50–1000 tons/10^6^ tons of processed crude oil) [18], while in China, VOC emissions from this industrial sector are estimated to range between 17.9% and 39.6% of the total emissions [41]. Critical sources of VOC emissions include petroleum product storage yards, maritime terminals, and (auto and rail) loading/unloading stations for petroleum products [42].

VOC emissions quickly disperse in the environment, where they persist for a long or short period depending on the climatic conditions. VOCs react with other pollutants (such as NO_x_) in the presence of light. Therefore, they are the primary precursors of the tropospheric ozone and particles in suspension (the smog’s main compounds). During the reactions in which they participate, VOCs can form organic compounds with higher molecular weight, which condense and produce secondary organic aerosol [43]. In these circumstances, VOC emission control has become a benchmark in European air quality monitoring programs [44].

Various interpolation methods have been utilized to evaluate the concentrations of gaseous compounds in the atmosphere at different locations. For example, Choi and Chong [45] proposed a modified version of the inverse distance weighting (IDW) method and conducted the pollutants zoning for 45 monitoring stations. Their results show significant improvement compared to those presented by Kianisadr et al. [46] for the pollutants in Khorramabad, Iran. The concentrations of VOC species typically exhibit substantial temporal and spatial variations. Hong et al. [47] studied the BTEX accumulation on road surfaces encompassing typical commercial, industrial, and residential lands using multiple linear and nonlinear regression and ANNs.

In Romania, special attention is paid to aligning with the EU Directive on air quality. Therefore, the study of atmospheric pollution (as a first step in taking measures to diminish it based on informed insight) and its impact on population health has become a topic of interest for scientists [6,7,8,9,48,49,50,51].

This research’s aim is twofold: (1) to assess VOC pollution levels from the second-biggest refinery in Romania and (2) to evaluate the noncarcinogenic and carcinogenic risks of benzene and TVOC exposure to the workers. We investigated the exceedance of maximum admissible values to achieve the first goal. The Peak Over Threshold method was utilized to model the maximum concentration series and determine the return periods. Given the high return levels for short return periods, extending the research to the second goal was found to be necessary.

The combined approach used here has the advantage of providing the expected highest pollution levels and correlating the results with the expected impact on public health. From another point of view, since high pollution could lead to a drastic decrease in tourism in the studied zone (the refinery is located in the neighborhood of the most renowned resorts on the Romanian Seaside), the study raises an alarm signal to the authorities to take urgent measures to reduce human and economic damages.

## 2. Study area and Methodology

### 2.1. Study Site

**Figure 1:**
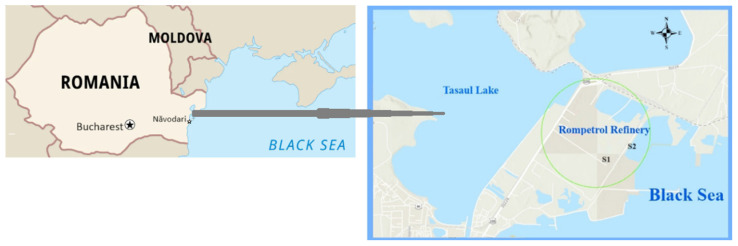
The map of Romania and the study area. Station 1 (S1)—44°20′06.96″ N, 28°38′26.40″ E (Loading/unloading auto ramp area); Station 2—44°20′19.01″ N, 28°39′20.94″ E (Loading/unloading railway ramp). The green circle represents the Refinery area.

Data were gathered from two monitoring stations (Figure 1) at the largest refinery in Romania and one of the most advanced in southeastern Europe. The oil refining complex is located on DJ 226, at Km 23, in Constanta County. The industrial platform (44°19′58″–44°20′55.7″ N and 28°38′13.3″–28°41′01.14″ E, respectively) is situated within the perimeter of the town of Năvodari, on the southern shore of the Tașaul Lake and the isthmus between it and the Siutghiol Lake. The main settlements in the area are the village of Corbu (2.5 km to the north), the town of Năvodari (3 km to the west-southwest), the village of Mamaia (4 km to the southwest), the resort of Mamaia (10 km to the south), and the city of Constanța (17 km to the south) [52].

**Figure 2:**
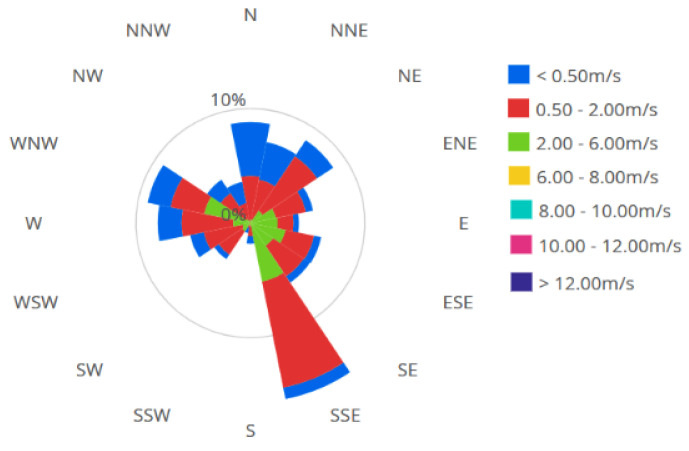
The windrose.

The region has a continental climate with sea influence, manifested through alternating daytime and nighttime breezes. The annual average temperature is about 11.2 °C. The annual average humidity (approximately 81%) is determined by continuous seawater evaporation, a factor that prevents excessive heating during the summer. The annual precipitation is below 400 mm. The wind rose is presented in Figure 2.

Considering the need for suitable storage space for both intermediate and final petroleum products and the distribution of petroleum products through loading into railway wagons, vehicles, and maritime terminals, the area experiences VOC emissions at concentrations that may exceed the legislatively prescribed upper limit values, the alert threshold for human health, and the critical level for the protection of vegetation and fauna, depending on weather conditions.

### 2.2. Data Series

The studied dataset is formed of the benzene and TVOC daily and monthly series collected during January 2021–December 2022 at Site 1 (44°20′06.96″ N, 28°38′26.40″ E)—the loading/unloading auto ramp area—and Site 2 (44°20′19.01″ N, 28°39′20.94″ E)—the loading/unloading railway ramp. These sites were chosen for study given that research from other countries indicated high concentrations of TVOC in such areas, as will be discussed in Section 3.4.

**Figure 3:**
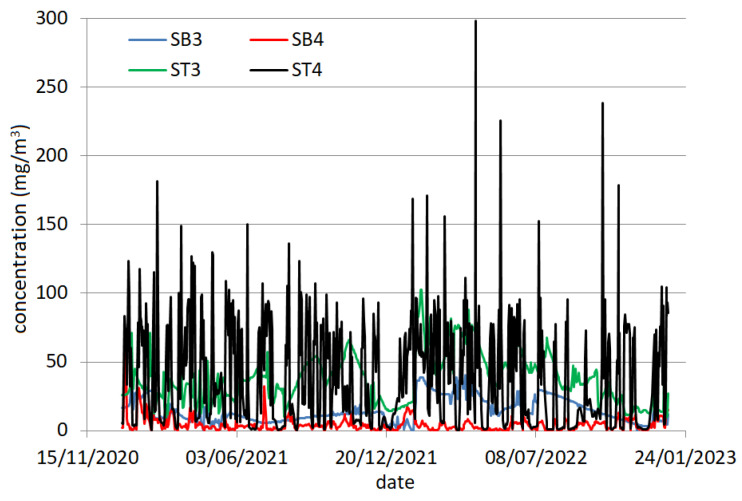
The series SB3, SB4, ST3, and ST4.

The monthly (daily) benzene series recorded at Site 1 and Site 2 will be denoted by SB1 (SB3) and SB2 (SB4), respectively. Similarly, the monthly (daily) TVOC series registered at Site 1 and Site 2 will be denoted by TB1 (TB3) and TB2 (ST4), respectively. The daily series are represented in Figure 3.

The sniffing method was used to measure the fugitive emissions of VOCs emanating from process equipment [53]. Accordingly, any detector type is permitted (e.g., based on catalytic oxidation, infrared absorption, flame ionization, or photoionization), provided it adheres to the specifications and performance criteria outlined in the standard. Additionally, EN 15446:2008 Standard outlines a procedure for estimating the emission rate from individual sources and the total emissions of the installation over a specified reporting period (typically a year) through a set of correlations [1].

The on-site air quality monitoring was carried out by the Best Available Techniques for Refineries (BATs) requirements as per Directive 2010/75/EU [54]. The quantitative analysis of the recorded data was conducted based on the limit values for the protection of human health (annual limit of benzene immission—5 μg/Nm^3^) imposed by Romanian Law no. 104/2011 [55] on environmental quality, the Directive 2001/81/EC [56], and Directive 2010/75/EU [54]. The Romanian Law no. 264/2017 [57] specifies that the hourly average concentration of vapors discharged from the vapor recovery unit, with the necessary correction applied for the dilution occurring during the process, must not exceed 35g/Nm^3^/h. Moreover, emission levels associated with BAT for emissions directed by VOC into the air should have a daily average or an average over the sampling period for total volatile organic carbon in the air in the range of 1–20 mg/Nm^3^ [58].

In the operation of gasoline loading and unloading facilities in tanks at terminals, the minimum requirements are: (1) the total measurement error of the calibration gas mixture must not exceed 10% of the measured value, and (2) the measurement equipment used must be capable of measuring concentrations of at least 3 g/Nm^3^ and have an accuracy of at least 95% of the measured value [59].

The VOC concentration series were obtained through fixed sensors: (1) AIT-102 (Ionscience Titan model), with the monitor consisting of a GC column and photoionization detector used for measuring the ambient concentration of benzene with internal data logging and (2) SD-D58-AC/DC (Riken Keiki model), with a continuous-monitoring detector head TVOCs providing a 4–20 mA signal indicating the target gas reading for use by a gas monitoring controller, recording device, or programmable controller, respectively. Mobile laboratories equipped with a modern air emissions analysis system (VOC analyzer with FID detection, Thermo Environmental Instruments model) and a meteorological station connected to a PC were utilized. The meteorological station enables online visualization of the registered concentration values.

Generally, the atmospheric evaporation profile of gasoline indicates high proportions of VOCs during the summer. Therefore, chemical compositions with Reid vapor pressure (RVP) ranging between 45 and 60 kPa are formulated in the warm season, while compositions with RVP values between 60 and 90 kPa are prepared in the cold season, depending on the volatility class of the petroleum product. However, due to meteorological phenomena, significant modifications may occur regarding fugitive emissions of VOCs into the atmosphere.

### 2.3. Data Analysis

The dataset was subject to statistical analysis to determine its characteristics and the similarities between the series recorded simultaneously at different locations. The steps followed were:

(1) Compute the basic statistics (mean, minimum, maximum, coefficient of variation, and skewness) and plot the histograms and boxplots to determine the series characteristics and emphasize the shapes of the series distribution and possible outliers.
(2) Apply the Anderson–Darling (AD) test [60] to test the hypothesis that the series is Gaussian against the hypothesis that the series is not normally distributed.
(3) Apply the Fligner–Killeen (KF) test [61] to check the homoskedasticity of each time series. The null (alternative) hypothesis is that the series is homoskedastic (heteroskedastic). The choice of this nonparametric test was based on the research of Conover et al. [62], which shows that this test is better than the alternatives in terms of power and when the normality hypothesis is not satisfied.
(4) Apply the Mann–Kendall (MK) [63] test to check the hypothesis that the series is random against the existence of a monotonic trend. When the null hypothesis was rejected, Sen’s [64] procedure was used to compute the slope of a linear trend.
(5) Apply the KPSS test [65] to test the null hypothesis of the series trend (or level) stationarity against its nonstationarity.
(6) Test the hypothesis that the series has no change points (breakpoint) against the hypothesis that it has at least one by performing the Buishand [66], Pettitt [67], Lee and Heghinian [68] tests, and Hubert segmentation procedure [69]. A change point appears when the series changes the mean, variance, or distribution from which it arose. The first three tests can determine only the most probable breakpoint. Moreover, the Buishand and Lee and Heghinian tests can be performed only if the series is Gaussian. If the series fails the normality test but can reach it by a transformation, the changepoint tests are run on the transformed series; otherwise, only the Pettitt and Hubert procedures can be performed. KhronoStat 1.01 (Hydrosciences Montpellier, France) [70] was utilized to conduct the tests.
(7) Apply the Kruskal–Wallis (K-W) test [71] to test if the series in a group originate from the same distribution against the alternative that at least one comes from a different distribution. When the null hypothesis was rejected, the post-hoc Dunn’s test [72], with the adjustment proposed by Hochberg [73], was run.

All tests were performed at a significance level α= 5%. A *p*-value less than 0.05 computed in a test (except Dunn’s) leads to rejecting the corresponding null hypothesis. In Dunn’s test, the null hypothesis is rejected if the *p*-value < α/2.

### 2.4. Modeling the VOC Series

According to Lenox and Haimes [74], extreme events have a low probability of apparition and high consequences. Extreme value theory is a statistical approach that focuses on the behavior of the probability distribution tails, describing the probabilities and magnitudes of extreme events. This method can help model people’s risks and exposures to high levels of pollutants that may pose acute or chronic health risks in the short and long term. These exposures have the most significant health impact and require accurate modeling [75].

For the series with the highest variability and showing mostly exceedances of the legal pollution limit, a Generalized Pareto Distribution (GPD) [76] was fitted using the Peak Over Threshold method (POT). POT models are employed when observations in a time series with high values, compared to the others, exceed a threshold. It was proved that these models are efficient when using sets (sometimes relatively small) of extreme values [77].

GPD has the following distribution function:

(1) $$G_{\xi ,\beta }(x)=\left\{\begin{array}{l} 1-{\left(1+\frac{\xi }{\beta }x\right)}^{-1/\xi },\;\;\xi \neq 0\\ 1-\exp\left(-\frac{x}{\beta }\right)\;\;\;\;,\;\;\xi =0 \end{array}\right.,\;$$

where β>0 and ξ are the scale and shape parameters, respectively. The *x* > 0 when ξ≥0, and 0<x≤−β/ξ when ξ<0.

Depending on the shape parameter’s value (ξ>0, ξ=0, or ξ<0), a particular distribution is obtained (Pareto (reparameterized), exponential, or Pareto type II).

Denote by $N_{u}$ the number of exceedances of a threshold *u*, *F* the distribution function of a random variable, and $F_{u}$ the distribution function of exceedances above *u* [78]:

(2) $$F_{u}\left(y\right)=\frac{F\left(y+u\right)-F(u)}{1-F(u)}.$$

It was shown that for a large class of distributions, when the threshold *u* increases, $F_{u}$ converges to a generalized Pareto distribution [79]. We work here in the hypothesis that if a pollutant series follows a theoretical distribution, *F*, and there is a threshold, *u*, then $F_{u}$(*y*) = ${G_{\xi ,\beta }}_{u}$. Maximum Likelihood Estimation [80,81] was utilized to estimate β and ξ. The threshold *u* is the lowest value for which the estimated ξ and $\beta_{u}=\beta -u\xi$ ($\hat{\xi }$,${\hat{\beta }}_{u}$) are almost constant in the plots of $\hat{\xi }$ and ${\hat{\beta }}_{u}$.

Using the fitted models, the return levels for different period units are also reported. In the case of POT, the return period *T* is expressed as a function of the probability distribution function *F* and the average waiting time between two extreme events, $\mu_{T},$ [82,83]:

(3) $$T=\frac{\mu_{T}}{1-F(x)}\;.$$

The return value is defined as a value that is expected to be equaled or exceeded on average once every interval of time *T* (with a probability of 1/*T*) [84].

The return period is useful for risk analysis. An extended return period indicates a low probability that an extreme value of a hazard will occur in any selected period (in our case, month or day).

The R packages extRemes and ismev were utilized for modeling.

### 2.5. Health Risk Assessment

The noncarcinogenic risk associated with VOCs is estimated by the hazard index (HI) for benzene and TVOC.

To assess the noncarcinogenic risk to workers in the industrial park from inhalation of VOCs, we computed HI for professionals in the categories: (1) Young, aged 18–25, with <5 years of experience, (2) aged 25–35, with 5–10 years of experience, (3) Experienced, aged 35–50, with 10–15 years of experience, (4) Seniors, aged 40–65, with 15–25 years of experience, using the equation [22,85,86]:

(4) $$\mathrm{HI}=\frac{C_{s\;}\times ET\times EF\times ED}{365\times {AT}_{nc}\times 24}\times \frac{1}{{RfC}_{s}}$$

where:

- $C_{s\;}$ (μg/m^3^) is the average daily concentration of VOCs;
- ET is the daily exposure time, considered 8 h/day for all workers;
- *EF* is the exposure frequency. *EF* has the following values function of the worker categories: (1) 350 days/year, (2) 340 days/year, (3) 325 days/year, (4) 340 days/year.
- *ED* is the exposure duration. *ED* has the following values function of the worker categories: (1) 5 years, (2) 10 years, (3) 15 years, (4) 25 years.
- ${AT}_{nc}$ is the average exposure time to noncarcinogenic risk, estimated at 74.8 years;
- ${RfC}_{s}$ (μg/m^3^) represents the reference concentration of VOC species for the noncarcinogenic risk assessment.

The values of ${RfC}_{s}$ are presented in Table 1. In the computation of HI for benzene, ${RfC}_{s}=$ 30 μg/m^3^, whereas for TVOC, ${RfC}_{s}=$ 1000 μg/m^3^, computed as a weighted average of the values from column 2 with the weight from column 4 of Table 1.

HI < 1 indicates the absence of potential noncarcinogenic health risks, while an HI exceeding 1 indicates the presence of potential chronic noncarcinogenic health risks.

We also computed the carcinogenic risk by Lifetime Cancer Risk (LCR) for the same categories of workers according to the equation:

(5) $$\mathrm{LCR}=\frac{C_{s\;}\times ET\times EF\times ED}{365\times {AT}_{c}\times 24}\times {IUR}_{s}$$

where:

- $C_{s\;}$, *ET*, *EF*, and *ED* have the same meaning and values as in Equation (3).
- ${AT}_{c}$ is the average time under exposure to carcinogenic risk, estimated at 70 years.
- ${IUR}_{s}$ represents the inhalation unit risk of VOC species for carcinogenic risk assessment. A cumulative value of ${IUR}_{s}$ = 2.5 × 10^−6^ (μg/m^3^)^−1^ was considered in the computation of LCR for TCOVs.

The permissible values for LCR are within the range of 10^−6^–10^−4^.

## 3. Results and Discussion

### 3.1. Results of the Statistical Analysis

**Figure 4:**
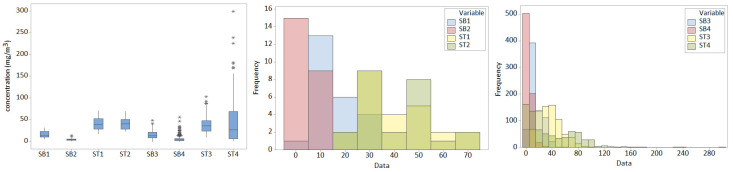
Boxplots and histograms of the data series.

Table 2 displays the basic statistics of the studied series. One can observe high variations of all but the SB2 series. The maximum TVOC concentrations are 102.76—ST3 and 298.58—ST4. ST4 and ST3 have the biggest standard deviations (stdev), indicating the highest dissipation around the series mean values. The highest coefficients of variation (cv) correspond to ST4, followed by SB4, confirming the high variability of these series. The skewness coefficients show that all series are right-skewed (the highest skewness being for SB4), confirmed by the histograms from Figure 4 (middle and right). SB4 is the series with the highest number of outliers (Figure 4, left).

Given the *p*-values lower than 0.05, the Anderson–Darling test (Table 3, row 2) rejected the normality hypothesis for all but ST1 and ST2 at a significance level of 5%. *p*-values (Table 3, row 3) less than 0.05 were obtained after running FK tests for all but SB2, ST1, and ST2, so there is enough evidence to reject the homoskedasticity hypothesis.

From a practical viewpoint, heteroskedasticity and non-normality are critical drawbacks when using different modeling techniques. Moreover, heteroskedasticity may indicate the series nonstationarity.

The MK test could not reject the randomness only for ST2 and ST3. Therefore, Sen’s slopes were estimated (row 5 of Table 3). The negative slopes of SB2, SB4, and ST4 indicate a decrease in the pollution trend. By contrast, the positive slopes of SB1, SB3, and ST1 indicate the augmentation of the pollution level (the highest for ST1, with a slope of 1.2596). The results indicate an inhomogeneous pollution trend at temporal and spatial scales that deserves further investigation in another study.

The KPSS rejected the series level stationarity for all but SB1, ST3, and ST4 (Table 2, row 5). The series trend stationarity was rejected only for SB3, SB4, and ST3 (Table 2, row 6). These findings confirm the results of the FK test, indicating that SB3, SB4, and ST3 have the highest variability, which makes it challenging to model and forecast the series evolution. For such an approach, stationarization is a must before modeling. The existence of change points in all series concurs with the KPSS test’s results.

**Figure 5:**
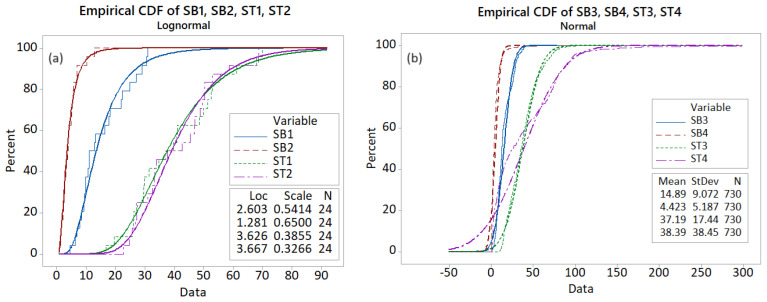
The Empirical and Theoretical Cumulative Distribution Functions—in the same color—for (**a**) SB1, SB2, ST1, and ST2; (**b**) SB3, SB4, ST3, and ST4.

The rejection of the null hypothesis K-W for the (SB1, SB2), (ST1, ST2), and (SB3, SB4) pairs indicates that the series in each pair did not come from the same underlying distribution. The Cumulative Distribution Functions (CDFs) represented in Figure 5, together with the theoretical curve—lognormal for the monthly series (a) and Gaussian for the daily series (b)—emphasize the series differences, confirming that the TVOC series have different patterns at different temporal and spatial scales.

### 3.2. Models for the Benzene and TVOC Series

**Figure 6:**
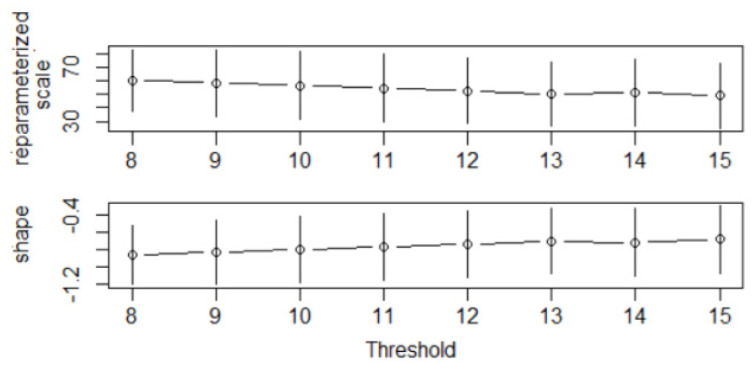
The threshrange plot containing the reparameterized scale and shape values, used for selecting the threshold in the model for ST3.

The first step in the POT is to determine the threshold above which the extreme values are further selected for building the models. The threshrange plot utilized in the case of ST3 is shown in Figure 6, and the thresholds in the POT models for all series are presented in Table 4, column 4. Columns 2 and 3 of the same table contain the estimated values ($\hat{\beta }$ and $\hat{\xi }$) of the scale and shape parameters.

All standard errors associated with $\hat{\xi }$ are under 0.3, whereas those associated with $\hat{\beta }$ are less than 1.35, indicating a good fit of the parameters (better for *ξ* than for *β*).

**Figure 7:**
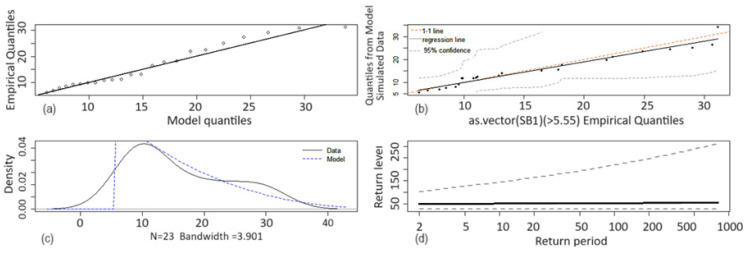
SB1 model: (**a**) QQ-plot; (**b**) QQplot2—Quantiles from Model Simulate Data vs. Empirical Quantile; (**c**) Data density plot and the model fitted density; (**d**) Return level plot.

**Figure 8:**
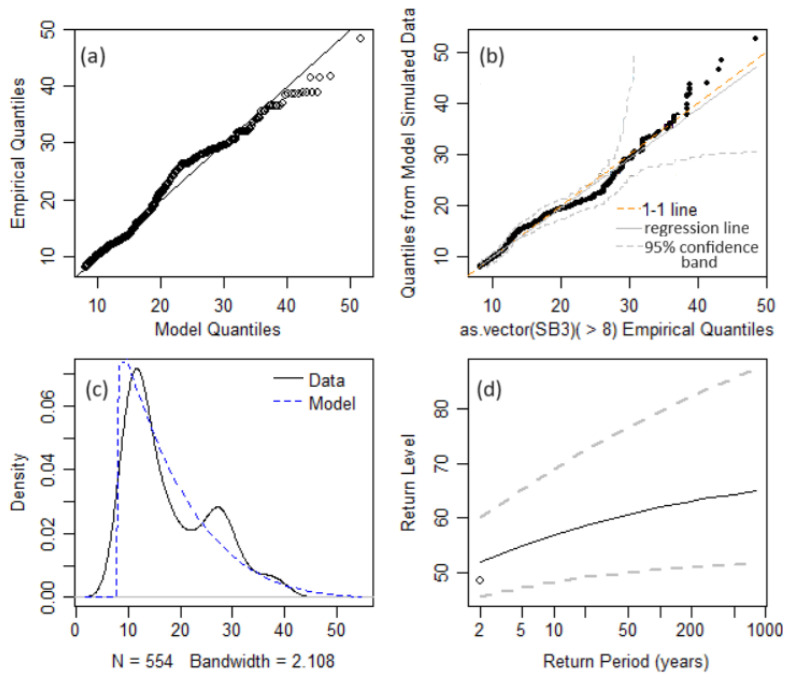
SB3 model: (**a**) QQ-plot; (**b**) QQplot2—Quantiles from Model Simulate Data vs. Empirical Quantile; (**c**) Data density plot and the model fitted density; (**d**) Return level.

The Anderson–Darling goodness of fit test validated the obtained models. The diagrams of the empirical quantiles vs. the model’s quantiles (Figure 7a and Figure 8b), theoretical density function vs. the data series (Figure 7c and Figure 8c), the chart of the quantiles from the model simulate data vs. empirical quantiles (Figure 7b) are provided to illustrate the goodness of fit of the models. Figure 7 and Figure 8 contain the mentioned charts for the SB1 and SB3. The dotted curves in Figure 7c,d and Figure 8c,d represent the limits of the confidence interval at the confidence level of 95%, and the continuous ones are the charts of the return level as a function of the return period.

In the case of a ‘perfect’ model, there should be a perfect match between the theoretical model and the data series (the dots Figure 7a,b and Figure 8a,b should be situated on the black lines).

The computed return levels for different return periods (2, 3, 4, 6, 12, 24 months for the monthly series and 2, 5, 10, 20, 50, and 100 days for the daily series) and are presented in Table 4, columns 5–10. All are above the maximum admissible levels established by legislation. The lowest return level corresponds to SB2 (between 20.09 and 27 μg/m^3^), whereas the highest is for ST4. In all cases, the return levels are very high, even those corresponding to high return periods, indicating alarming levels of atmospheric pollutants.

### 3.3. Health Risk Indicators

Table 5 contains the values of HI and LCR with respect to the VOC emissions from the studied sites.

Categ (1)–(4) are the categories of professionals according to Section 2.5. The HI values in italics exceed one, and the LCR values in bold are higher than the permissible limit. HI values are between 3.13 × 10^−2^ (TVOCs at Site 1) for a young professional with less than 5 years of work experience and 6.39 × 10^0^ (benzene at Site 2) for a senior professional with 15–25 years of experience. LCR values are between 8.38 × 10^−5^ (TVOCs at Site 1) for a young professional with under 5 years of work experience and 9.13 × 10^−4^ (TVOCs at Site 2) for a senior professional with 15–25 years of experience.

All HI values for benzene are above 1 at Site 2, and half are above 1 at Site 1, indicating a high noncarcinogenic risk; with respect to TVOC, HI > 1 only for the fourth category. Overall, 43.75% of HI values are above one, indicating a relatively high noncarcinogenic risk for benzene and TVOCs, predominantly at the loading/unloading railway ramp. Over 93.75% of LCR values exceed the upper admissible limit, meaning there is also a high carcinogenic risk for all considered categories of workers.

These findings align with the high recorded VOC concentrations, which are mainly over the admissible limits, during the entire study period.

### 3.4. Discussions

Many researchers have noted that volatile pollutants could accumulate in urban and suburban areas and roads near industrial oil refinery parks [2,4,5,11,20,39,41,88,89,90]. Zhang et al. [89] indicate that the emission rates of Chinese refineries have consistently exceeded those of certain developed countries with traditions in crude oil processing, including in European countries [2,5] and the northern and southern states of the USA. For example, the total annual emission (with dominant VOC species of aliphatic and aromatic hydrocarbons emitted in nearly all units) from an oil refinery in China’s Pearl River Delta region was around 12,600 kg [90]. The authors found that the spatial arrangement of VOCs revealed that 87.5% of airborne VOCs consisted of benzene, toluene, xylene, and ethylbenzene, with a higher concentration (146 μg/m^3^) observed in the northern oil refinery industrial areas. Toluene, benzene, and p-xylene concentrations reached 41.5, 33.3, and 79.7 μg/m^3^, respectively. Assessing the benzene dispersion during four seasons at a petroleum waste depot on Kharg Island (Iran), Karbasi et al. [91] estimated that about 1151 m^3^ of hydrocarbons evaporated annually from the surface of the terminal’s reservoirs. Wei et al. [92] indicated that during the research period (in a refinery in China), the TVOCs accumulated in the neighborhood increased by 61 ppb. A study from a refinery in Thailand [20] emphasized that the highest predicted concentrations of pentane exceeded the odor and ${RfC}_{s}$ threshold. Our findings are concordant with those of the mentioned studies.

In all cases, measures to reduce VOC emissions from refineries are necessary. Among the VOC emission sources (valves, flanges, single seals in pumps and compressors, and leaks in equipment and pipelines), valves contribute 50–60% of fugitive emissions. Therefore, the study of Saikomol [20] suggested adding a secondary seal to the floating roof of the oil storage tank, which would decrease the emission and concentration of pollution at the ground level. He also indicates this solution to be the cheapest from the point of view of the cost per unit of concentration reduction. Virdi et al. [93] emphasized the necessity of employing vapor recovery units and proved the practical results in their study.

Other measures must include petroleum product storage and loading areas, such as tanks for crude oil and light products, which must be supplied with double-sealed floating or fixed roofs. High-efficiency seals—including enhanced primary seals and additional secondary or tertiary seals (depending on existing losses)—are devices designed to limit vapor losses. Currently, these models are considered to be the best available techniques. Still, their use may be restricted when retrofitting tertiary seals in existing tanks. In addition, various techniques are applied in industrial processing units to reduce losses due to evaporation caused by the heating of stored chemical products.

The loading ramps must have automated loading systems and volatile recovery units. During venting operations, pump leaks, sampling for analysis, and water leaks from tanks, vessels, and boilers with products, strict supervision must be maintained to prevent excessive leakage, ensuring the flow of petroleum products into the appropriate collecting basin only.

Industrial installations and equipment must be regularly inspected and maintained to ensure proper functioning. Periodic integrity and tightness checks must be conducted to operate industrial machinery and equipment safely, including valves, pumps, pipelines, tanks, pressure vessels, vessels for capturing drops, and hazard warning systems. Moreover, the people who work on the platform must be equipped with protective masks during specific operations.

## 4. Conclusions

This article proposes the analysis of the TVOC series collected in one of the most important refineries in Romania and the impact of pollution from VOCs on workers at the refinery. This study emphasized a high variability of the emission series, with most of the monthly and daily recorded values above the permissible limits. Some series have negative trends with very low slopes, so it is impossible to conclude that there is an accentuated tendency for pollution decrease. Moreover, most have at least a breakpoint and are trend- or level-nonstationary, indicating a high variability in VOC emissions. These contrasting results should be further investigated after sampling from other points and correlating with other variables.

The POT analysis found high return values for all return levels, indicating an elevated degree of pollution. Therefore, urgent measures must be taken to determine the causes of the high recorded VOC concentrations in the atmosphere and control the emissions from the studied industrial plant. Some involve employing vapor recovery units or double seals for floating roof storage of the tanks to avoid atmospheric contamination. Periodic inspection of the installation and the loading/unloading ramps and immediate measures to eliminate or reduce possible oil leakage are also necessary.

A comprehensive action plan must be designed after deeply analyzing each factor contributing to atmospheric pollution. This study will be presented in a further article. Implementation of such a plan is crucial to ensure adequate air quality for workers and nearby populations in areas with high potential risks.

## Figures and Tables

**Table 1:** VOC species, ${RfC}_{s}$, ${IUR}_{s}$, and the percentages in TVOC [85,87].

VOC Species	${RfC}_{s}$ (μg/m^3^)	${IUR}_{s}$ (μg/m^3^)^−1^	Percentage
benzene(C_6_H_6_)	30	7.8 × 10^−6^	37.00
toluene (C_7_H_8_)	5000	-	14.00
ethylbenzene (C_8_H_10_)	1000	2.5 × 10^−6^	7.50
styrene (C_8_H_10_)	1000	-	
m-,p-xylene (C_8_H_10_)	100	-	8.30
o-xylene (C_8_H_10_)	100	-	1.80
1,2,3-trimethylbenzene (C_9_H_12_)	60	-	3.00
n-hexane (C_6_H_14_)	700	-	28.00
cyclohexane (C_6_H_12_)	6000	-	0.10

**Table 2:** Basic statistics for benzene and TVOCs series.

Statistics	SB1	SB2	SB3	SB4	ST1	ST2	ST3	ST4
mean	15.50	4.36	14.89	4.42	40.26	41.16	37.19	38.39
stdev	8.27	2.84	9.07	5.19	14.96	13.43	17.44	38.54
minimum	4.61	0.80	0.00	0.00	16.80	22.84	9.78	0.42
maximum	31.13	12.96	48.45	56.00	69.97	68.87	102.76	298.58
cv (%)	53.35	65.13	60.92	117.28	37.17	32.63	46.88	101.15
skewness	0.69	1.52	0.84	3.69	0.43	0.53	0.69	1.47

**Table 3:** The *p*-values in the AD, FK, MK, and KPSS tests. The values inside brackets are Sen’slopes. Blank space means that the slope is not significant.

p-val	SB1	SB2	SB3	SB4	ST1	ST2	ST3	ST4
p-val AD	0.0111	0.0207	0.0000	0.0000	0.2564	0.1450	0.0000	0.0000
p-val FK	0.0421	0.5093	0.0000	0.0000	0.1202	0.4720	0.0000	0.0001
p-val MK/ (Sen’slope)	0.0395/ (0.4775)	0.0161/ (−0.1916)	0.0013/ (0.0049)	0.0040/ (−0.001)	0.0106/ (1.2596)	0.2059	0.4415	0.0000/ (−0.0151)
p-val KPSS-level	0.0825	0.0464	0.0100	0.0100	0.0336	0.1000	0.0100	0.0407
p-val KPSS-trend	0.0691	0.0823	0.0100	0.0100	0.100	0.1000	0.0100	0.1000

**Table 4:** Results of the GPD models.

**Series**	$\hat{\beta }$	$\hat{\xi }$	**Threshold**		**Return Period**				
				**2**	**3**	**4**	**6**	**12**	**24**
SB1	13.2525	−0.2714	5.55	46.13	46.99	47.55	48.26	49.31	50.18
SB2	2.7860	0.0005	5.55	20.09	21.22	22.02	23.16	25.09	27.03
ST1	42.8420	−0.7807	16.2	70.75	70.84	70.89	70.94	71.00	71.03
ST2	39.9693	−0.8323	19.35	67.18	67.23	67.26	67.30	67.33	67.35
**Series**	$\hat{\beta }$	$\hat{\xi }$	**Threshold**		**Return Period**				
				**2**	**5**	**10**	**20**	**50**	**100**
SB3	12.8678	−0.2331	8	51.82	54.89	56.89	59.49	60.64	61.94
SB4	3.7235	0.2069	4.25	43.61	55.59	66.28	78.62	97.90	115.12
ST3	38.4456	−0.6441	17.40	76.15	76.57	76.76	76.88	76.97	77.02
ST4	71.4874	−0.5441	15.00	141.54	143.44	144.36	145.00	145.54	145.81

**Table 5:** The maximum values of HI and LCR with respect to Benzene and TVOC.

Species	Site	Health Index	Categ (1)	Categ (2)	Categ (3)	Categ (4)
Benzene	1	HI	4.93 × 10^−1^	8.38 × 10^−1^	*1.20 × 10^0^*	*1.84 × 10^0^*
		LCR	**1.23 × 10^−4^**	**2.09 × 10^−4^**	**3.00 × 10^−4^**	**4.62 × 10^−4^**
	2	HI	*1.70 × 10^0^*	*2.89 × 10^0^*	*4.15 × 10^0^*	*6.39 × 10^0^*
		LCR	**1.42 × 10^−4^**	**2.42 × 10^−4^**	**3.47 × 10^−4^**	**5.34 × 10^−4^**
TVOCs	1	HI	3.13 × 10^−2^	5.33 × 10^−2^	7.64 × 10^−2^	1.17 × 10^−1^
		LCR	8.38 × 10^−5^	1.42 × 10^−4^	2.04 × 10^−4^	3.14 × 10^−4^
	2	HI	2.73 × 10^−1^	4.64 × 10^−1^	6.65 × 10^−1^	*1.02 × 10^0^*
		LCR	**2.43 × 10^−4^**	**4.14 × 10^−4^**	**5.93 × 10^−4^**	**9.13 × 10^−4^**

## Data Availability

Data will be available on request from the authors.
